# Molecular Characterization of Complete Genome Sequence of an Avian Coronavirus Identified in a Backyard Chicken from Tanzania

**DOI:** 10.3390/genes14101852

**Published:** 2023-09-23

**Authors:** Henry M. Kariithi, Jeremy D. Volkening, Gaspar H. Chiwanga, Iryna V. Goraichuk, Peter L. M. Msoffe, David L. Suarez

**Affiliations:** 1 Exotic and Emerging Avian Viral Diseases Research Unit, Southeast Poultry Research Laboratory, U.S. National Poultry Research Center, Agricultural Research Service, USDA, Athens, GA 30605, USA; 2 Biotechnology Research Institute, Kenya Agricultural and Livestock Research Organization, P.O. Box 57811, Nairobi 00200, Kenya; 3 BASE_2_BIO, Oshkosh, WI 54904, USA; 4 Tanzania Veterinary Laboratory Agency, South Zone, Mtwara P.O. Box 186, Tanzania; 5 National Scientific Center Institute of Experimental and Clinical Veterinary Medicine, 61023 Kharkiv, Ukraine; 6 Department of Veterinary Medicine and Public Health, Sokoine University of Agriculture, Chuo Kikuu, Morogoro P.O. Box 3021, Tanzania; 7 National Ranching Company Ltd., Dodoma P.O. Box 1819, Tanzania

**Keywords:** AvCoV, lineage GI-19, LX4/QX-type IBV, oropharyngeal swab, recombination

## Abstract

A complete genome sequence of an avian coronavirus (AvCoV; 27,663 bp excluding 3′ poly(A) tail) was determined using nontargeted next-generation sequencing (NGS) of an oropharyngeal swab from a backyard chicken in a live bird market in Arusha, Tanzania. The open reading frames (ORFs) of the Tanzanian strain TZ/CA127/19 are organized as typical of gammaCoVs (*Coronaviridae* family): 5′UTR-[ORFs 1a/1b encoding replicase complex (Rep1ab) non-structural peptides nsp2-16]-[spike (S) protein]-[ORFs 3a/3b]-[small envelop (E) protein]-[membrane (M) protein]-[ORFs 4a/4c]-[ORFs 5a/5b]-[nucleocapsid (N) protein]-[ORF6b]-3′UTR. The structural (S, E, M and N) and Rep1ab proteins of TZ/CA127/19 contain features typically conserved in AvCoVs, including the cleavage sites and functional motifs in Rep1ab and S. Its genome backbone (non-spike region) is closest to Asian GI-7 and GI-19 infectious bronchitis viruses (IBVs) with 87.2–89.7% nucleotide (nt) identities, but it has a S gene closest (98.9% nt identity) to the recombinant strain ck/CN/ahysx-1/16. Its 3a, 3b E and 4c sequences are closest to the duck CoV strain DK/GD/27/14 at 99.43%, 100%, 99.65% and 99.38% nt identities, respectively. Whereas its S gene phylogenetically cluster with North American TCoVs and French guineafowl COVs, all other viral genes group monophyletically with Eurasian GI-7/GI-19 IBVs and Chinese recombinant AvCoVs. Detection of a 4445 nt-long recombinant fragment with breakpoints at positions 19,961 and 24,405 (C- and N-terminus of nsp16 and E, respectively) strongly suggested that TZ/CA127/19 acquired its genome backbone from an LX4-type (GI-19) field strain via recombination with an unknown AvCoV. This is the first report of AvCoV in Tanzania and leaves unanswered the questions of its emergence and the biological significance.

## 1. Introduction

The avian coronavirus (AvCoV) infectious bronchitis virus (IBV; genus *Gammacoronavirus*, family *Coronaviridae* [1]) causes contagious upper respiratory, enteric and urogenital disease in poultry depending on factors such as the breed of the infected bird and vial strain and tissue tropism [2]. In addition to IBVs, AvCoVs infecting turkeys (TCoVs), pheasants (PhCoVs), ducks (DuCoVs), geese (GoCoVs), pigeons (PiCoVs) and guineafowls (GfCoVs) fall within the GammaCoVs group [3,4]. Both IBVs and TCoVs cause substantial economic losses in chicken and turkey industries worldwide and are the most extensively studied AvCoVs. Following its first description and isolation in chickens in the U.S. in the 1930s, IBV has been identified globally and in a wide host range including chickens, turkeys, pheasant and other *Galliformes* species such as quail and ornamental birds [5,6]. Similarly, after the initial identification of TCoVs in poults in the 1970s in the U.S. [7], TCoVs and TCoV-like variants have been detected in turkey and chicken flocks across North and South America (e.g., Canada and Brazil) and Eurasian countries (e.g., France, Italy, Poland, UK and China) [8,9].

AvCoVs have a linear plus-strand (+) RNA genome (27.3–31.3 kb) comprising multiple reading frames (ORFs) flanked by 5′-/3′-unstranslated regions (UTRs). Gene 1 has two overlapping ORFs (ORF1a/b) occupying 5′-proximal two-thirds of the genome (~20 kb) encoding viral replicase complex polypeptide (Rep1a/b) comprising up to16 non-structural proteins (named sequentially as nsp1-nsp16), which are co-/post-translationally processed by virally encoded main (3CL ^pro^; nsp5) and accessory (PL ^pro^; nsp3) proteinases via proteolytic cleavage [10]. The 3CL ^pro^ self-processes itself from Rep1ab, and subsequently cleaves the polypeptide at 11 conserved sites with consensus motif x-[L/I/V/F/M]-Q↓[A/S/G], where “x” is any residue and “↓” is the cleavage site [11]. The 3′-proximal one-third of the genome downstream of gene 1 contains the structural spike (S), envelop (E), membrane (M) and nucleocapsid (N) proteins encoded by genes 2, 3, 4 and 6, respectively, and accessory non-structural proteins encoded by genes 3 (3a and 3b), 4 (4b and 4c), 5 (5a and 5b) and 6 (6b) [12,13,14]. AvCoV genomes are typically ordered as 5′UTR-[Rep1ab]-[S]-[3a-3b-E)]-[M-4a-4c]-[5a-5b]-[N-6b]-3′UTR-poly(A) tail, with the genes named after their products. Upon polyadenylation at the 3′-end, the viral genome serves as mRNA template for the Rep1ab complex during genome replication [1,15].

The spike protein is the largest of the structural proteins and is post-translationally cleaved by cellular proteases into subunits S1 and S2; S1 harbors serotype-specific antigenic epitopes associated with the hypervariable regions (HVRs) [16]. For IBVs, the S1 heterogeneity, which results from mutations and/or recombination events, distinguishes at least 32 distinct lineages within eight genotypes (GI to G-VIII), and dozens of unique variants (UVs) and inter-lineage recombinants that do not classify with the established lineages [17,18]. However, this system, which relies on the full-length S1 sequence, is limited because the vast majority of strains only have partial S1 sequences. Emergence of novel variants is largely attributable to within-/between-species recombination events, which occur naturally at recombination breakpoint hot-spots, some of which are conserved [19]. The main recombination mechanism (i.e., “copy choice”) occurs during genome replication when RNA-dependent RNA (RdRp; encoded by nsp12) detaches from the viral RNA template being copied and then re-attaches at a recombination breakpoint in a homologous position of a different RNA template, thus resulting in a recombinant strain derived from two different parental strains [20]. Such recombination events are implicated in the emergence of TCoVs [21,22]; one of the consequences of these events in TCoVs is that they are able to infect and replicate in chickens, but without inducing the severe enteric disease observed in turkeys [4,21,23].

Currently, only eight GI lineages (GI-1, GI-12, GI-13, GI-14, GI-16, GI-19, GI-23 and GI-26) and several UVs have been reported in Africa [18], but little is known about their impacts on the poultry industry in the continent. The current study reports the complete genome sequence of a recombinant TCoV-like strain we identified using nontargeted next-generation sequencing (NGS) of clinical samples collected during a Newcastle disease viruses (NDVs) surveillance study in backyard chickens presented for sale at live bird markets (LBMs) in Tanzania. This is the first report of the presence of AvCoV in Tanzania and should contribute to future investigations into these viruses and vaccine options for disease control in the country and east African region.

## 2. Materials and Methods

### 2.1. Samples

The samples analyzed in the current study were collected from adult backyard chickens using standard procedures during a surveillance of NDVs conducted between September 2018 and May 2019 at LBMs in six regions in Tanzania—Arusha, Dar es Salaam, Iringa, Mbeya, Morogoro and Tanga. Briefly, an oropharyngeal (OP) and cloacal (CL) swab sample was collected from each bird, then stored in individual 2.0 mL cryogenic vials (Corning Inc., New York, NY, USA) containing 1.5 mL of Difco™ brain-heart-infusion broth (Thermo Fisher, Waltham, MA, USA) and immediately stored in liquid nitrogen during the sampling exercise and transportation from the field to the laboratory, where they were preserved at −80 °C at Sokoine University of Agriculture in Tanzania until they were shipped to Southeast Poultry Research Laboratory (SEPRL), USDA-ARS in Athens, GA, USA for analyses. At the time of sampling, vaccination status or histories of the sampled birds were not available, and clinical signs consistent with avian diseases were recorded.

### 2.2. Total RNA Extraction and Next-Generation Sequencing

Total RNAs were extracted separately from OP and CL samples using MagMAX™-96 AI/ND Viral RNA kit and pre-treated with an in-house RNaseH rRNA depletion protocol, as previously described [24]. For NDV surveillance, previously described real-time reverse transcription-polymerase chain reaction (rRT-PCR) L-/M-tests [25,26] were used to detect the virus in the pre-treated RNAs. Then, samples from 20 birds containing high amounts of NDV RNAs by the rRT-PCT tests (cycle threshold (*C*_T_) cutoff of below 30) were randomly selected for the NGS. Sequencing libraries were prepared from the selected samples using sequence-independent, single-primer amplification [27] and Illumina Nextera TM Flex protocols, followed by paired-end sequencing (600-cycle MiSeq Reagent Kit v3) on an Illumina MiSeq instrument, as previously described [18].

### 2.3. Genome Sequence Assembly, Annotation and Characterization

Raw NGS data were analyzed using a nontargeted taxonomic classification and assembly pipeline (BASE₂BIO LLC; Oshkosh, WI, USA), which involved removal of residual sequencing adaptors, primers and host-specific reads using Trim Galore v0.6.7 (github.com/FelixKrueger/TrimGalore (accessed on 18 January 2023)) and BBTools (https://sourceforge.net/projects/bbmap/ (accessed on 18 January 2023)) and final sequence assembly using MEGAHIT v1.2.9 [28] with default parameters. Quality of the assembly was assessed using BWA-MEM [29] and coverage/artifacts inspected using IGV [30]. The assembled consensus sequences were annotated using Geneious Prime^®^ v2023.1.2 (www.geneious.com (accessed on 18 January 2023)), as recently described [31,32]. Sequences from this study and other AvCoVs (retrieved from GenBank using BLASTn algorithm) were aligned using MAFFT v7.490 [33], trimmed using trimAl v1.2 [34] and used for Maximum Likelihood phylogenetic analysis in MEGA 11 (1000 bootstrap replicates) [35]. Recombination events were assessed using Recombination Detection Program v4.101 (RDP4) as described previously [32,36]. Genome-wide comparative pairwise homology was performed and visualized using ClustVis [37].

### 2.4. Sanger Sequencing

A recently described Sanger sequencing protocol [18] was used to determine missing bases in ORF1a of the assembled consensus genome sequence (*n* = 8 nt at position 11,064–11,071) with primers specifically designed for the gap, i.e., IBV-10886F (5′-CTC CTA TGC GGA GTA CGA A-3′) and IBV-11529R (5′-CAC TGC AGC GTA GAG TCT-3′).

## 3. Results

### 3.1. NGS and Sequence Assembly

AvCoV-specific RNA was detected in an OP sample collected from a chicken (bird ID: CA127) at an LBM in Arusha. The NGS produced 26,485 read pairs specific for IBV, which were assembled de novo into a contiguous sequence (one contig) 27,663 nt in length with a median coverage depth of 262 reads, but with an 8 nt gap in Rep1a gene (position 11,064–11,071), which was successfully filled using Sanger sequencing (Table 1). The complete genome described here is named AvCoV/ck/TZ/2145-CA127/19 (hereafter abbreviated as TZ/CA127) and assigned GenBank accession number OQ725698. As shown in Table 1, NGS detected RNAs of other microbial agents of avian interest including viruses (NDV, chicken megrivirus (ChMeV) and chicken avastrovirus (CAstV)), and bacteria (*Enterococcus* sp., *Avibacterium paragallinarum*, *Mycoplasma synoviae*, *Ornithobacterium rhinotracheale* and *Riemerella anatipestifer*. The CL sample of bird CA127 contained a few paired reads specific for AvCoV (*n* = 281) and NDV (*n* = 618), which could be assembled into short contigs (~400–800 nt in length) that matched to various viral genes.

### 3.2. Genome Organization and Classification of the Tanzanian AvCoV

**Figure 1:**
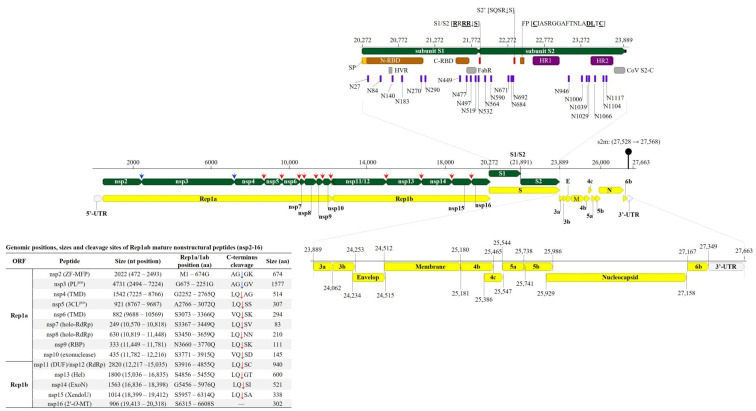
Schematic representation of the genome organization of Tanzanian AvCoV strain TZ/CA127 identified in the current study. The yellow, green and gray bars represent the coding sequences of the viral genes (CDS), mature peptides of replicase 1ab (Rep1ab) and spike (S) genes and 5′-/3′-untranslated regions (UTRs), respectively. The blue and red downward arrows (↓) in the middle panel and in the table (bottom left) indicate the proteolytic cleavage sites processed by 3C-like main proteinase (PL ^pro^) and papain-like proteinase (3CL ^pro^), respectively, to produce the nonstructural peptides (nsp2-16) of Rep1ab (the 11 3CL ^pro^ sites are conserved in CoVs sites [3]). The structural features/domains found in subunits S1 and S2 of the spike gene are highlighted, including the N- and C-terminal receptor-binding domains (N-/C-RBD), hypervariable region (HVR), antigen-binding region (FabR), primary (S1/S2) and auxiliary (S2′) cleavage sites, fusion peptide (FP), heptad repeat regions (HR1 and HR2), CoV C-terminal cysteine-rich intravirion region (CoV-S2-C) and *N*-linked glycosylation sites (*n* = 23, numbered using aa positions relative to the first methionine (M) residue of the S protein). The underlined amino acid residues in the S gene are conserved in CoVs and the black downward arrow (↓) indicates cleavage site. Abbreviations: DUF, protein domain of unknown function; ExoN, 3′-to-5′ exoribonuclease; Hel, helicase; holo-RdRp, RNA-dependent RNA polymerase (RdRp) holoenzyme; XendoU, poly(U)-specific endoribonuclease; PL ^pro^, papain-like protease; RBP, RNA-binding protein; Rep1a/1b, Replicase 1a/1b; 3CL ^pro^, 3C-like cysteine protease; TMD, transmembrane domain; 2′-*O*-MT, *S*-adenosylmethionine-dependent ribose 2′-*O*-methyltransferase; ZF-MFP, zinc-finger multifunctional protein; s2m, stem-loop 2 motif.

**Figure 2:**
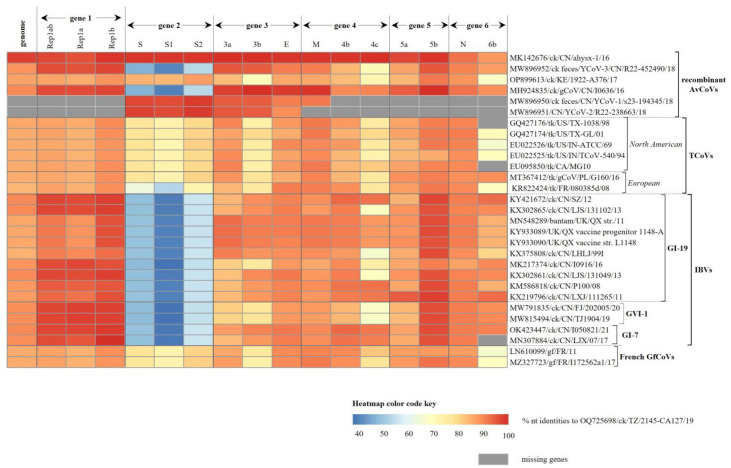
Comparative pairwise identities across the genome of the Tanzanian strain TZ/CA127 and other AvCoVs. The color of the heatmap changes from blue to blood orange with increasing nucleotide identities; gray boxes indicate missing genes. Overall, the TZ/CA127 genome is similar to the Chinese strain ahysx-1/16 and other Asian recombinant AvCoVs. Sequence names include GenBank accession number, abbreviated avian host species, 2-letter country abbreviation, strain name and year of isolation/reporting. Complete comparative nucleotide identities across the genome are presented in Table S1. Abbreviations: AvCoV, avian coronavirus; GfCoV, guineafowl coronavirus; IBV, infectious bronchitis virus; TCoV, turkey coronavirus; GI, lineage I.

Consistent with other AvCoVs, the TZ/CA127 genome sequence is organized as follows: 5′UTR-Gene 1 (Rep1ab)-Gene 2 (S)-Gene 3 (3a, 3b and E)-Gene 4 (M, 4a and 4c)-Gene 5 (5a and 5b)-Gene 6 (N and 6b)-3′UTR (Figure 1). Its 5′-UTR is 471 nt long with a higher GC content (50.4%) compared to the whole genome sequence (38.2%), while the 314 nt long 3′-UTR contains a 41 nt long stem-loop-2 motif (s2m; position 27,528–27,568), a highly conserved element in viruses with (+) ssRNA genomes [38]. As shown in Table 2, the complementary transcription-regulating sequences (TRSs) are located at varying distances from the gene start codons, and have the conserved core sequence AACAA consistent with CoVs [39]. Overall, the genome is most similar (97.52% nt identity) to the Chinese strain ck/CN/ahysx-1/16 (GenBank accession number MK142676), a recombinant AvCoV (Figure 2; Table S1). Compared to strains belonging to the established IBV lineages, the genome of TZ/CA127 showed highest nt identities (87.2–89.7% range) to Asian GI-19 and nephropathogenic GI-7 IBVs (formerly QX-/LX4-like and TW-I/II-like, respectively [17]).

#### 3.2.1. Structural Protein Genes

**Figure 3:**
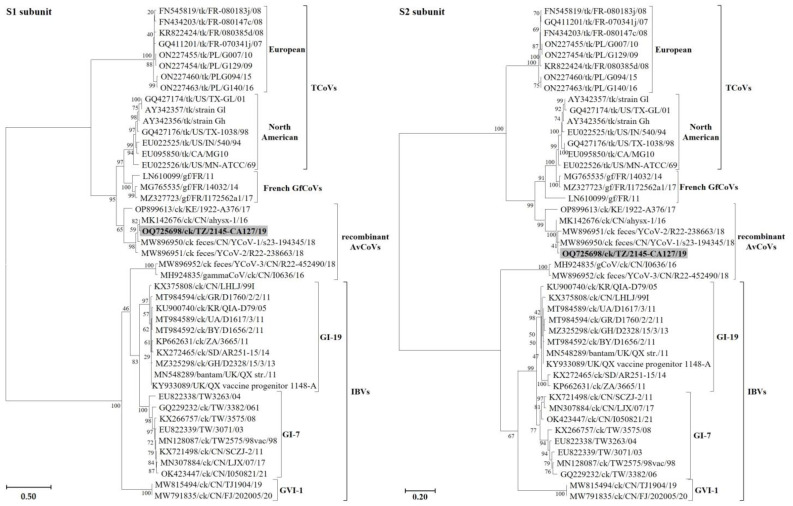
Phylogenetic relationship of the Tanzanian strain TZ/CA127 (highlighted in gray) with other AvCoVs based on nucleotide sequences of the S1 and S2 subunits. The final dataset used in the analysis involved 45 sequences and 1462 and1861 positions for the S1 and S2 sequences, respectively. Sequence names include GenBank accession number, abbreviated avian host species, 2-letter country abbreviation, strain name and year of isolation/reporting. Abbreviations: AvCoV, avian coronavirus; GfCoV, guineafowl coronavirus; IBV, infectious bronchitis virus; TCoV, turkey coronavirus; GI, lineage I.

Consistent with other CoVs, the 3′-proximal one-third of the TZ/CA127 genome sequence is characterized by the structural genes S, E, M and N (Figure 1; Table 2). The length of its S gene sequence (3618 nt) is comparable to its homologs in TCoVs (size range of ~3600–3629 nt), which is longer than the typical size of IBVs (~3498 nt). The complete S gene sequence is closest to chicken origin Chinese strains ahysx-1 (98.9%), Yunnan CoV-1 (97.48%) and Yunnan CoV-2 (97.60%). The sequence similarity drops considerably with the next closest match being North American TCoVs and French GfCoVs with nt identities in the range of 76.5–77.2% (complete S), 71.8–73.3% (subunit S1) and 79.4–80.9% (subunit S2)—Table S1. The nt identities of the TZ/CA127 S sequence to GI-7 and GI-19 IBVs are in the low ranges of 48–49% (complete S), 37.5–39.8% (subunit S1) and 54.8–56.1% (subunit S2). Phylogenetically, both the S1 and S2 nt sequences placed TZ/CA127 with the strains ahysx-1 and Yunnan CoV-1/-2 in a larger cluster containing the North American TCoVs and French GfCoVs (Figure 3; see detailed tree in Figure S1).

**Figure 4:**
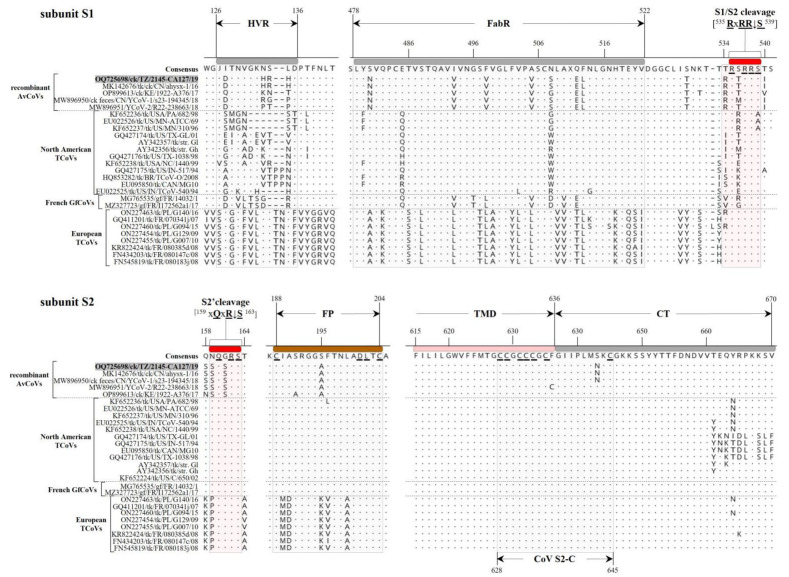
Amino acid (aa) variations in the domains of subunits S1 and S2 of the Tanzanian strain TZ/CA127 (highlighted in gray) compared to other AvCoVs. The domains highlighted include the hypervariable region (HVR), antigen-binding region (FabR), S1/S2 cleavage site (RxRR↓S) and auxiliary S2′ cleavage motif (xQxR↓S) where “x” is any aa residue and “↓” is the cleavage position, transmembrane domain (TMD), C-terminal cysteine-rich intravirion region (CoV-S2-C; shown at the bottom of the S2 sequence alignment) and cytoplasmic tail (CT). Residues in bold and underlined in the consensus sequence are conserved in CoVs (see text). The dots and dashes in the alignments indicate identical and missing (or gaps in alignment of) aa residues, respectively. The aa residues in the consensus are numbered from the beginning of each subunit in the aligned sequences, i.e., the first methionine and serine residues immediately following the S1/S2 cleavage site (RxRR↓S) for subunits S1 and S2, respectively. Sequence names include GenBank accession number, abbreviated avian host species, 2-letter country abbreviation, strain name and year of isolation/reporting. Abbreviations: AvCoV, avian coronavirus; GfCoV, guineafowl coronavirus; TCoV, turkey coronavirus.

The main domains of the S protein sequence (i.e., conserved or functionally relevant [8,12,40,41]) present in TZ/CA127 are presented in Figure 1. They include N-/C-terminal receptor-binding and antigen-binding (FabR) domains, S1/S2 and auxiliary S2′ cleavage motifs (R**R**x**RR**↓**S** and S**Q**S**R**↓**S**, respectively; conserved residues are underlined and “↓’ is cleavage site), membrane fusion peptide (FP; consensus sequence **C**IASRGGAFTNLA**DL**T**C**; conserved residues are underlined), heptad repeat regions 1 and 2 (HR1/2), *N*-linked glycosylation sites (*n* = 23) and C-terminal cysteine-rich intravirion region (CoV-S2-C). Rather than the three hypervariable regions (HVRs I-III) found in IBVs, only one HVR is present in the TZ/CA127 subunit S1 sequence, which is consistent with TCoVs [8,42]. Figure 4 presents alignment of S1 and S2 sequences of TZ/CA127 and other AvCoVs in the regions containing some the key domains of the two subunits, i.e., the HVR, FabR, S1/S2 and S2′ cleavage sites, FP and CoV-S2-C. Note that the results presented in Figure 4 only include viruses in the four groups that cluster most closely with TZ/CA127 in Figure 3, i.e., recombinant TCoVs, North American TCoVs and European TCoVs and French GfCoVs. The transmembrane anchor (cytoplasmic tail) that contains the 18 nt-long CoV-S2-C domain is highly conserved across the analyzed sequences, and as expected, the HVR has many aa variations (mutations, insertions/deletions) when compared to other analyzed sequences. Overall, the alignment largely distinguishes the viruses into the above-mentioned four groups.

The TZ/CA127 E gene sequence is similar in length (282 nt) to its homologs in ahysx-1 and gammaCoV/I0636 (GenBank accession MH924835), which is shorter than the average lengths of IBV S sequences (324–333 nt size range). As shown in Table S1, the TZ/CA127 E sequence is closest (99.65% nt identity) to a duck strain DuCoV/GD/27/14 (GenBank accession number NC_048214)—a dominant CoV in duck flocks in Guangdong province, China [43]. The lengths of TZ/CA127 M (669 nt) and N protein genes (1230 nt) are consistent with other AvCoVs, which is expected because they are among the most conserved. As described in other CoVs [12], the M protein (222 aa) has three transmembrane domains (aa residues 15–37, 44–66 and 76–98), while the N protein (409 aa) has RNA-binding (aa residues 6–165) and dimerization (aa residues 218–325) domains. Compared to other AvCoVs (Table S1), both the M and N sequences of the Tanzanian virus were most similar to their homologs in strains ahysx-1 (99.25% and 92.44% nt identities, respectively) and gammaCoV/I0636 (99.10% and 91.95% nt identities, respectively). Phylogenetically, the E and M sequences monophyletically cluster TZ/CA127 with the Chinese recombinant AvCoVs and the Eurasian GI-7/GI-19 IBVs, but the N gene appears distinct (Figure S2). Contrary to the three structural genes, and with a topology similar to that of the spike region, complete genome sequences of the Tanzanian, Chinese (CN/ahysx-1/16) and the Kenyan (KE/1922-A376/17) strains group with the TCoVs/GfCoVs away from the GI-7/GI-19 viruses and gammaCoV/I0636 and Yunnan-CoV-3 strains (compare Figure 4 and Figure S2).

#### 3.2.2. Non-Structural Protein Genes

AvCoVs have two sets of nonstructural proteins—the Rep1ab peptides encoded by gene 1 (nsp2-16) upstream of the spike region and the small accessory products of gene 3 (3a and 3b), gene 4 (4b and 4c), gene 5 (5a and 5b) and gene 6 (6b) that are found interspersed downstream of the spike region among the structural genes [12,44]. The architecture of these genes as annotated for the TZ/CA127 genome in the current study is illustrated in Figure 1.

The features of Rep1ab (ORF1ab) in the TZ/CA127 genome are typical of AvCoVs, including its size (19,851 nt; position 472–20,321), conservation of the heptanucleotide slippery sequence UUUAAAC (position 12,252–12,258) at which Rep1a (11,817 nt) and Rep1b (ORF1b; 8037 nt) peptides are produced via the ribosomal frameshifting (RFS) mechanism [45] and the proteolytic cleavage sites (consensus sequence x-[L/I/V/F/M]-Q↓[A/S/G] where “x” is any residue and “↓” is the cleavage site) that produce nsp2-16 [11,46]. As summarized in Table S2, the sizes of nine out of the 14 nsps are conserved across all analyzed sequences, i.e., nsp5, nsp7-10, nsp11/12 and nsp14-16. All 14 cleavage sites of the recombinant AvCoVs and GI-7/GI-19IBVs are 100% identical, except nsp10 (exonuclease) of the Kenyan strain, which has isoleucine and alanine residues (***I***Q↓S***A***) compared to valine and aspartate residues (***V***Q↓S***D***) in the other analyzed sequences. We further analyzed nsp12 (RdRp) because it is the most functionally important of the Rep1ab nsps (genome replication/transcription). As expected of RNA viruses, there was high conservation of the seven RdRp motifs known for RNA viruses (typically organized as G-F-A-B-C-D-E [47]), except aa variations in motifs G and A (Figure S3). In motif G, the Tanzanian/Chinese recombinant AvCoVs and the UK/Chinese GI-7/GI-19 IBVs have phenylalanine (F500) compared to tyrosine (Y500), while in motif A, the recombinant AvCoVs and IBV GI-7 strains have methionine (M608) compared to isoleucine (I608) in other analyzed sequences. All the RdRp functionally critical aa residues such as those involved in interactions with the RdRp cofactors (i.e., nsp7 and nsp8 holoenzymes), RNA-binding and catalytic activities are strictly conserved across the analyzed sequences, except in motif C, where the North American strain TCoV-540 has alanine (A754) compared to serine (S754) in other analyzed strains. Because the RdRp has been suggested as an alternate to the S1-based IBV characterization [48], we performed phylogenic analysis using the translated aa sequences of the nsp12, which monophyletically clustered the Tanzanian strain with the Chinese recombinant AvCoVs and the GI-7/GI-19 IBVs (Figure S4).

The sizes of the TZ/CA127 accessory genes (3a and 3b (174 and 192 nt, respectively), 4b and 4c (285 and 162 nt, respectively), 5a and 5b (198 and 249 nt, respectively) and 6b (195 nt)) are generally consistent with their homologs in other CoVs [12,49]. ORFs 3a, 3b and 4c are most similar to the Chinese chicken and duck strains ahysx-1/16 and DuCoV/GD/27/14, with nt identities in the range of 99.3–100% (Table S1). The monophyletic clustering of the Tanzanian virus and GI-7/GI-19 strains observed in the structural genes was also observed in the nonstructural gene sequences, except in the 3a/b sequences of GI-7 strains (Figure S5). Further, 6b sequence of the Polish GI-19 strain PL/G160/16, which reportedly emerged from recombination between GI-19 IBV and a North American TCoV-/French GfCoV-related strain [50], clusters with the AvCoVs/IBVs away from its TCoV counterparts. Overall, with the exception of the Rep1a and 6b genes, the nonstructural gene sequences of the Tanzanian and ahysx-1 strains cluster together; this topology is also evident in the phylogenetic trees based on the complete genome, and the structural genes E, M and subunit S1 (compare Figure 1 and Figure S2).

### 3.3. Recombination Events in Tanzanian AvCoV

**Figure 5:**
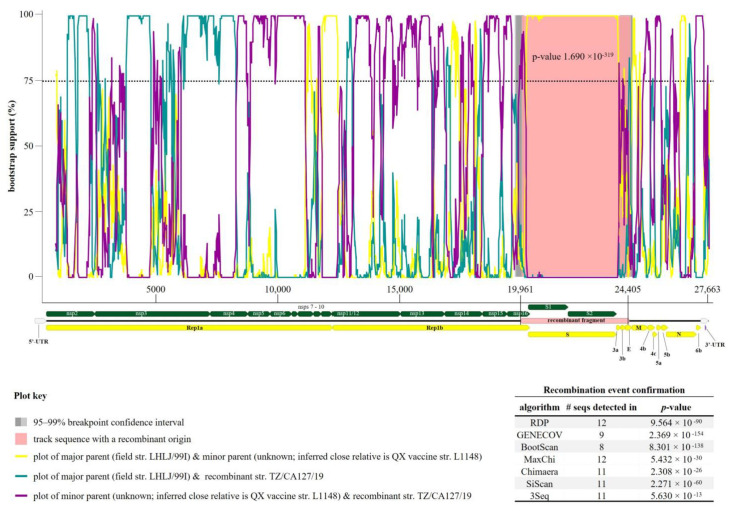
Recombination event signal detected in the Tanzanian strain TZ/CA127/19 reported in the current study supported by seven algorithms of RDP4. The “major parent” (i.e., the strain most identical to the sequence surrounding the recombination breakpoints) was predicted to be GI-19 IBV strain LHLJ/99I (GenBank accession numbers KX375808), and the sequence of the unknown “minor parent” (i.e., the strain most identical to the transferred sequence fragment) was inferred from QX vaccine strain L1148 (GenBank accession KY933090). The recombinant fragment is illustrated by the rose-colored area surrounded by the recombination breakpoints (nt position 19,961–24,405). The recombination was supported by seven out of the nine RDP4 algorithms as tabulated at the bottom right of the figure. The genome map of TZ/CA127/19 is shown on the X-axis of the plot (drawn to scale); the yellow, green and gray bars on the represent the coding sequences of the viral genes (CDS), mature peptides of replicase 1ab (Rep1ab) and spike (S) genes and 5′-/3′-untranslated regions (UTRs) of the genome sequence, respectively. The analysis involved 69 full genome sequences of recombinant AvCoV (*n* = 9), TCoV (*n* = 12), GfCoV (*n* = 3), DuCoV (*n* = 1) and IBV GI-7 (*n* = 5), GI-19 (*n* = 39) viruses. The criterion for the selection of the sequences used for the recombination signal detection was based on the results obtained from the genome sequence and phylogenetic analyses as presented in this study.

The results obtained from the sequence and phylogenetic analyses presented above suggested that the Tanzanian strain could be a recombinant. Seven out of the nine RDP4 algorithms identified a recombination event with the breakpoints beginning at position 19,961 (C-terminus of nsp16) and ending at position 24,405 (N-terminus of E gene) of the TZ/CA127 genome sequence (Figure 5). From the de novo genome assembly, the recombination site was noted to have deep reads coverage (median depth coverage of 447) as illustrated in Figure S6.

An LX4-type (GI-19) strain LHLJ/99I (GenBank accession: KX375808) is the predicted major parent, i.e., the strain closest to the sequence surrounding the recombination breakpoints (91.2% nt identity). However, the sequence of the minor parent (meaning the strain most identical to the recombinant fragment transferred to the Tanzanian strain) remained unknown, but its inferred close relative is a commercial GI-19 attenuated vaccine strain L1148 (GenBank accession number KY933090), a derivative of QX vaccine progenitor strain 1148-A [51]. The recombination event detected in TZ/CA127 is located in one of the main recombination breakpoint hot-spots known in CoVs (i.e., approximately 800 nt upstream of the S gene, which can result in the transfer of the entire spike region from the donor to the acceptor RNA genome templates [19]). Our analysis gave indication that the predicted major parental strain (LHLJ/99I) is also likely a recombinant (three recombination signals were predicted in its S, 3b/E and N/6b gene regions) with the major parent being a GI-19 IBV (LX4-type; strain ck/CH/LSD/03I) and an unknown minor parent (inferred close relative is either North American TCoV/TX-1038 or French GfCoV/I172562a2). However, the three recombination event signals detected in strain LHLJ/99I were insufficiently supported by RDP4 because of at least one of the following: unverifiable beginning/ending of breakpoints, supported by less than five algorithms, one or both parental strains predicted as possible actual recombinants [36]. Another recombinant fragment was detected in the Tanzanian strain TZ/CA127/19—predicted major parent is Kenyan strain KE/1922-A376/17 (97.2% nt identity) and minor parent is gammaCoV strain I0636/16 (99.2% nt identity). The recombination breakpoints of the 247 nt long recombinant fragment are located within nsp3 (PL ^pro^) of Rep1a at position 2780–3026; it was confirmed by seven RDP4 algorithms, with a *p*-value of 1.608^−22^. Seven other recombination signals (*n* = 5 in the Rep1ab, and one each in genes 4 (M and 4b) and 6 (N and part of 6b) were also detected in the Tanzanian strain, but they were disqualified for insufficient RDP4 support based on the criteria described above in the case of strain LHLJ/99I.

## 4. Discussion

Backyard chickens in sub-Saharan Africa account for over 80% of the poultry flocks comprising mixed species (i.e., crosses or exotic chicken varieties, ducks, geese, turkeys, etc.), which are reared under small-scale free-range or semi-extensive system for food security and income source [52,53,54]. These so-called “village flocks” (i.e., multiple flocks from neighboring households and villages) are to a large extent predisposed to infectious viral pathogens because of, among other factors, inadequate veterinary services, nutrition, shelter, biosecurity and their frequent interactions with synanthropic wild birds as they scavenge for food [55]. The situation is further aggravated by the largely unregulated live poultry trade where middlemen buy the birds from several villages at a time and transport them to various traders at the LBMs, where they kept in wire-mesh cases under poor conditions for several days before they are sold out (stock turnover is mostly biweekly) where they are slaughtered for food or purchased for return to other farms. These scenarios present an ideal avenue for infectious agents to invade broader geographical regions where they can evolve into novel variants, especially in the case of mutation-prone contagious RNA viruses such as IBVs [56,57].

The above-mentioned situations hold true in the Tanzanian backyard poultry farming, and, like most of sub-Saharan Africa, the country lags behind countries in other continents in terms of availability of AvCoV sequence data and information on impacts of infectious bronchitis disease (IB) on backyard poultry where the disease is mainly found compared to commercial poultry farming [58]. Further, there is currently no proper surveillance or vaccination program for IBVs in Tanzania, and no documented evidence of the presence of AvCoVs in the country. The Tanzanian AvCoV strain TZ/CA127/19 reported in the current study was an incidental discovery through the use of random non-targeted NGS of swab samples collected from backyard chickens during NDV surveillance in the country. NDV is considered to be the most important poultry pathogen in the Tanzanian poultry industry [55,59], and it is no surprise that we also identified a virulent NDV genotype VII.2 strain as a coinfection with strain TZ/CA127/19. It should be noted that the NGS was performed on samples from only 0.98% of the birds that were sampled for the NDV surveillance (*n* = 20 out of a total of 20,494 birds). Thus, the finding of a complete AvCoV genome sequence in 5% of the NGS-tested samples (1 out of 20) should precipitate more surveillance efforts and epidemiological investigations because there is high likelihood of the presence of more AvCoV variants, perhaps with high prevalence.

Like in other members of the genus *Gammacoronavirus* [1], the 27.7 kb genome of strain TZ/CA127/19 has 5′- and 3-proximal UTRs of sizes within the expected range of 200–600 nt (i.e., 471 nt and 314 nt, respectively) flanking the six ORFs that are architecturally conserved in CoVs in the order of: ORFs 1a and 1b (encoding the replicase complex proteins; nsp2-16) and four ORFs encoding the structural proteins S, E, M and N. Additionally, between the structural genes, its genome has small ORFs encoding the accessory proteins 3a/3b (between S and E genes), 4b/4c and 5a/5b (between M and N genes) and 6b (between N gene and the 3′-UTR). We did not detect reads covering the 3′-proximal poly(A) tail region, but this is rather common because the homopolymeric nature of poly(A) tails is one of the challenges of whole-genome NGS. The structural genes of strain TZ/CA127/19 contain features that are conserved across AvCoVs such as the cleavage sites in the Rep1ab (and the RFS site) and the spike genes, functional motifs in the aa sequences of the RdRp and S glycoprotein, TRSs upstream the gene start codons, s2m in the 3′-UTR, etc. The conservation of these and other features potentially implies that the replication and virion assembly of the Tanzanian strain are governed in a manner similar to other CoVs. Unlike the well-characterized S, E, M, N and Rep1ab genes [12], many aspects of the accessory genes (3a/b, 4b/c, 5a/b and 6b) are only speculated, including their origin (e.g., acquired horizontally from their avian host species), the mechanism of their expression (standard vs nonstandard translation), their roles in viral pathobiology (e.g., functionally essential vs “junk’ genes). However, they have been reported in IBVs and TCoVs, and although dispensable for the in vitro viral replication, some of them (e.g., 3a/b, 4b, 5a/b) are expressed during viral infection [12,49,60,61,62].

Collectively, our data strongly suggest that TZ/CA127/19 acquired most of its genome backbone (non-spike region) from an Asian GI-19 IBV through recombination at breakpoints located at one of the main CoV recombination hot-spots [63]. Notable, GI-19 viruses are amongst the topmost four widely distributed IBVs (others are GI-1 (Mass-type), GI-13 (793B or 4/91) and GI-16 (Q1)); they were recognized as a distinct Chinese genotype in the 1990s, and subsequently spread to Russia, Europe, the Middle East and Africa (Egypt, Zimbabwe and South Africa) between early 2000 and 2015 [17,56,64,65]. In Africa, GI-19 IBVs have been reported in the northern (Algeria and South Sudan), western (Ghana and Nigeria) and southern (South Africa and Zimbabwe) regions of the continent [18]. Another aspect to note is the relatively high identity of the Tanzanian virus to the Asian GI-7 IBVs, and their monophyletic clustering with GI-19 IBVs. Like the GI-19, GI-7 IBVs were also first identified in China in the 1990s and subsequently became the third-most prevalent genotype in the country, but there is no documented evidence of their detection outside of China and Taiwan [17,66,67]. Further, there are reports of recombination between GI-7 and GI-19 genotypes, as well as between the two genotypes and field/vaccine strains belonging to different genotypes [68,69]. This is an important aspect to consider because such recombination can rapidly and unpredictably generate novel variants with adaptive capabilities such as for host shifting and altered tissue tropism and antigenicity/pathogenesis [21].

The TZ/CA127/19 S gene is comparable to, but genetically distinct from, the S gene of TCoVs, as demonstrated by the shared nt similarities (~76–77%) compared to less than 50% to GI-7 and GI-19, which is expected based on available literature [21,70]. Considering the TCoV-like nature of the spike region, one would expect the minor parent of the Tanzanian strain to be a TCoV strain, which is not the case—rather, the unknown minor parent was inferred to be a close relative of a Chinese commercial QX-like attenuated vaccine (strain L1148). The likelihood of vaccine strains being involved in the emergence and evolution of the Tanzanian strain cannot be totally ruled out because continued exposure of poultry to live vaccines is among the factors thought to contribute to natural recombination of IBVs [71], which is even more likely in high density poultry flocks with mixed species. However, the types of IBV vaccine strains potentially present in Tanzanian poultry flocks are unknown, but this does not necessarily indicate the non-existence such strains. Further, there is currently no documented evidence of the existence of TCoVs in Tanzania or any of the neighboring Eastern and Central African countries, except our recent report of the Kenyan TCoV-like strain [18]. It is possible that the potential TCoV strain (or an intermediate/transient TCoV-like recombinant) that may have undergone recombination with a GI-19 strain to produce the mosaic TCoV-like spike region of the Tanzanian strain is yet to be identified.

One of the outstanding questions from the current study is how the potential parental GI-19 (or GI-7) virus could have spread from Asia to Tanzanian in eastern Africa. Among other factors, long-distance migratory birds are one of the speculative contributors to virus dissemination [65], especially considering that Tanzania lays along the East Africa-Asia flyway and other migration corridors with major stopover and wintering grounds where the migratory birds congregate [72]. These migratory birds interact with local wild birds, which at some point scavenge for food with the backyard “village poultry”. There is also the challenge of recombination events to the nomenclature and classification of AvCoVs, which is currently based on the full-length S1 subunit sequences [17]. Although robust, the S1-based classification excludes a vast majority of AvCoVs with only partial S1 sequences. Furthermore, because S1 is prone to high rates of point mutations and recombination, there is reduced probability of newly discovered variants fitting within the established lineages, which are referred currently to as “unique variants” (UVs) [17]. Our phylogenetic analyses based on the RdRp, which has been suggested as an alternate marker for characterization of IBVs [48], produced results consistent with those obtained from using the non-spike genomic regions. Although we did not include large datasets in our analyses, our data hint at the need to reexamine the criteria for the classification of these viruses. Finally, there is the challenge of assessing the biological and epidemiological significance of newly emerging variants (e.g., their pathogenicity, differentiation between infected and vaccinated poultry, etc.) and whether the currently used vaccines are potent in protecting domestic poultry from their infections; strain selection for vaccine programs are currently based the classical and variants IBVs from North America (Mass, Ark and Conn), Europe (793B, CR88 and D274), which elicit poor immune responses, and therefore they may contribute to the generation of novel variants [73].

## 5. Conclusions

We have presented for the first time the presence of an AvCoV in backyard poultry in Tanzania, and for the second time in East and Central Africa following our recent report of a recombinant AvCoV in the neighboring country (Kenya). The ability of the IBVs to widen their host range increases chances of recombination and the subsequent evolution and emergence of novel variants with even greater capabilities of transmission and infection, especially when mixed species of poultry are reared together. Although the current study was limited in terms of temporal, spatial and sample representation of the Tanzanian backyard poultry, the discovery of this variant in samples meant for surveillance of NDVs in LBM chickens adds to the limited repertoire of genetic information of AvCoVs in sub-Saharan Africa and are invaluable in future investigations into the epidemiology and control of IB.

## Figures and Tables

**Table 1:** Summary of data obtained from NGS of an OP swab sampled at an LBM in the urban district of Arusha in Tanzania.

NGS Read Pairs			De novo Genome Assembly			Number of Missing Bases			Other Microbial Pathogens of Avian Interest Detected with High Confidence (Specific Read Count) *	
Total	Host-Specific (%)	IBV-Specific	Median Coverage Depth (Reads)	Consensus Seq. (Contig) Length	Completeness (%)	5′-End	# Internal Gaps (Length; Position)	3′-end	Viral (Specific Reads)	Bacterial (Specific Reads)
510,552	8.41%	26,485	262	27,663	98.50%	42	8-nt; 11,064–11,071	0	vNDV-VII.2 (177,140); ChMeV (110); AAstV (101)	*E. faecium* (202); *ORT* (1638); *R. anatipestifer* (1143); *M. synoviae* (*n* = 953); *A. paragallinarum* (303)

* Abbreviation: AAstV, Avastrovirus; *A. paragallinarum*, *A. paragallinarum*, *Avibacterium paragallinarum*; ChMeV, chicken megrivirus; *E. faecium*, *Enterococcus faecium*; *M. synoviae*, *Mycoplasma synoviae*; ORT, *Ornithobacterium rhinotracheale*; *R. anatipestifer*, *Riemerella anatipestifer*; vNDV, virulent Newcastle disease virus.

**Table 2:** Genomic coordinates and features of the genes of the Tanzanian strain TZ/CA127 reported in the current study.

Gene	Reading Frame	Gene Product	Genomic Position	Nucleotide Length (aa)	Putative Transcription Regulatory Sequence (TRS)		
					Sequence ^a^	Position	TRS—Initiation Distance (nt) ^b^
5’-UTR	-	-	1–471	471	-	-	-
Gene 1	+1	Rep1a	472–12,288	11,817 (3938)	CTT**AACAA**	3–10	461
		Rep1ab	472–20,321	19,851 (6616)			
Gene 2	+1	S1	20,272–21,891	1620 (540)	CTG**AACAA**	20,212–20,219	52
		S2	21,892–23,889	1998 (665)			
Gene 3	+3	3a	23,889–24,062	174 (57)	CTG**AACAA**	23,858–23,865	23
	+2	3b	24,062–24,253	192 (63)			
	+3	E	24,234–24,515	282 (93)			
Gene 4	+2	M	24,512–25,180	669 (222)	CTT**AACAA**	24,450–24,457	54
	+2	4b	25,181–25,465	285 (94)			
	+3	4c	25,386–25,547	162 (53)			
Gene 5	+2	5a	25,544–25,741	198 (65)	AAC**AACAA**	25,481–25,488	55
	+1	5b	25,738–25,986	249 (82)			
Gene 6	+3	N	25,929–27,158	1230 (409)	CTT**AACAA**	25,828–25,835	93
	+2	6b	27,167–27,349	183 (60)	AGG**AACAA**	27,075–27,082	84
3’-UTR	-	-	27,350–27,663	314	-	-	-

^a^ The underlined motif (AACAA) is conserved in CoVs. ^b^ Distance between the putative TRS and the transcription start of the corresponding gene.

## Data Availability

The complete genome sequence of the Tanzanian strain AvCoV/ck/TZ/2145-CA127/19 reported in this paper has been deposited in GenBank under accession number OQ725698. Raw data were deposited in the SRA under accession number SRR23899253, BioSample number SAMN33770569 and BioProject number PRJNA945007.

## Supplementary Materials

The following supporting information can be downloaded at: https://www.mdpi.com/article/10.3390/genes14101852/s1, Figure S1: Phylogenetic clustering of the Tanzanian strain TZ/CA127/19 identified in the current study (highlighted in red bold font) and representatives of other AvCoVs based on the nucleotide sequences of the full-length S1 gene. The lineages are named based on the current IBV classification system and are color coded based on their geographical distribution [17,18]. The two unique variants (UVs) recently reported from Tanzania neighboring Kenya are highlighted in purple [18]. The phylogenetic reconstruction involved 129 sequences, with the final dataset having 1385 positions. Sequence names include GenBank accession number, abbreviated avian host species, 2-letter country abbreviation, strain name and year of isolation/reporting. Abbreviations: AvCoV, avian coronavirus; DuCoV, duck coronavirus; gCoV, γ coronavirus; GfCoV, guineafowl coronavirus; IBV, infectious bronchitis virus; TCoV, turkey coronavirus; GI-GVIII, lineages I-VIII; UV, unique variant. Figure S2: Phylogenetic relationship of the Tanzanian strain TZ/CA127 identified in the current study with other AvCoVs based on nucleotide sequences of the structural envelop (E), membrane (M) and nucleocapsid (N) genes and complete genomes. The analysis included 31 sequences and 26,818, 276, 666 and 1230 positions for the complete genome, E, M and N sequences, respectively. Sequence names include GenBank accession number, abbreviated avian host species, 2-letter country abbreviation, strain name and year of isolation/reporting. Abbreviations: AvCoV, avian coronavirus; GfCoV, guineafowl coronavirus; TCoV, turkey coronavirus; Figure S3. Alignment of the amino acid (aa) sequences of the nsp region that contains the seven RdRp motifs (organized as G-F-A-B-C-D-E) known for RNA viruses [46] comparing the Tanzanian strain reported in the current study (highlighted in red font) with other AvCoVs. The aa residues in the consensus are numbered from the first residue of nsp12. Functionally important aa residues are color coded on the consensus sequence (color code key at bottom right of the figure). The dots and dashes in the alignments indicate identical and missing (or gaps in alignment of) aa residues, respectively; Figure S4. (A) Phylogenetic tree of the translated amino acid sequence of nsp12 (RdRp) of the Tanzanian strain TZ/CA127/19 reported in the current study and (B) its homologies to other AvCoVs. The analysis involved 41 sequences and 926 positions; Figure S5. Phylogenetic relationship of the Tanzanian strain TZ/CA127 with other AvCoVs based on nucleotide sequences of the non-structural genes (Rep1a, Rep1b, 3a/b, 4b/c, 5a/b and 6b). The analysis included 31 sequences and 11,797, 7956, 358, 423, 442 and 222 positions for the complete genome, Rep1a, Rep1b, 3a/b, 4b/c, 5a/b and 6b sequences, respectively; Figure S5. Phylogenetic relationship of the Tanzanian strain TZ/CA127 identified in the current study with other AvCoVs based on nucleotide sequences of the non-structural genes (Rep1a, Rep1b, 3a/b, 4b/c, 5a/b and 6b). The analysis included 31 sequences and 11,797, 7956, 358, 423, 442 and 222 positions for the complete genome, Rep1a, Rep1b, 3a/b, 4b/c, 5a/b and 6b sequences, respectively. Sequence names include GenBank accession number, abbreviated avian host species, 2-letter country abbreviation, strain name and year of isolation/reporting. Abbreviations: AvCoV, avian coronavirus; GfCoV, guineafowl coronavirus; TCoV, turkey coronavirus; Figure S6. Coverage depth (paired reads) of the recombination site (highlighted in rose-colored box; genomic nucleotide position 19.961 to 24,405) in the de novo genome assembly of the of the Tanzanian strain TZ/CA127 identified in the current study in relation to the coverage depth of the rest of the genome. The genomic coordinates of the open reading frames (ORFs) and the 3′-/5′-untraslated regions (UTRs) are shown in orange and gray vertical bars, respectively at the top of the figure. Note that only a portion of the reads are shown in the figure; Table S1: Comparative pairwise nucleotide identities of the Tanzania TZ/2145-CA12719 reported in the current study and representative strains of other AvCoV genotypes. The highest and the lowest identities are highlighted in bold and italicized underlined bold texts, respectively. Sequence names include GenBank accession number, abbreviated avian host species, 2-letter country abbreviation, strain name and year of isolation/reporting. Dash (—) indicates missing genes. Abbreviations: AvCoV, avian coronavirus; DuCoV, duck coronavirus; GfCoV, guineafowl coronavirus; IBV, infectious bronchitis virus; TCoV, turkey coronavirus; GI, lineage I. Table S2: Sizes (nucleotides) of Rep1ab mature peptides and the consensus sequences of the proteolytic cleavage sites of the Tanzanian strain TZ/CA127/19 reported in the current study (highlighted in bold red font) compared to other AvCoVs. The consensus sequence x-[L/I/V/F/M]-Q↓[A/S/G], where “x” is any residue and “↓” is the cleavage site [11]. Variations in the consensus sequences are highlighted (bold underlined). Sequence names include GenBank accession number, abbreviated avian host species, 2-letter country abbreviation, strain name and year of isolation/reporting. Abbreviations: rec. AvCoV, recombinant avian coronavirus; GfCoV, guineafowl coronavirus; IBV, infectious bronchitis virus; TCoV, turkey coronavirus; GI, lineage I; DUF, protein domain of unknown function; ExoN, 3′-to-5′ exoribonuclease; Hel, helicase; holo-RdRp, RNA-dependent RNA polymerase (RdRp) holoenzyme; XendoU, poly(U)-specific endoribonuclease; PL ^pro^, papain-like protease; RBP, RNA-binding protein; Rep1a/1b, Replicase 1a/1b; 3CL ^pro^, 3C-like cysteine protease; TMD, transmembrane domain; 2′-*O*-MT, *S*-adenosylmethionine-dependent ribose 2′-*O*-methyltransferase; ZF-MFP, zinc-finger multifunctional protein.
