# Supplementary material for: Molecular Characterization of Complete Genome Sequence of an Avian Coronavirus Identified in a Backyard Chicken from Tanzania
Source: Genes (Basel). 2023 Sep 23;14(10):1852. doi: 10.3390/genes14101852 (PMC10606662; doi:10.3390/genes14101852)
Supplement: Supplementary file 1 [file genes-14-01852-s001.zip › genes-2607752/mdpi-disclosure-form_hmk_GHC-1.pdf]

Multidisciplinary
Digital Publishing
Institute

MDPI AG
St Alban-Anlage 66
CH–4052 Basel
Switzerland

Tel. +41 61 683 77 34
Fax +41 61 302 89 18
www.mdpi.com

Disclosure of Potential Conflicts of Interest

All authors must disclose all relationships or interests that could inappropriately influence or bias their work. Although the presence of a relationship or activity does not always indicate a problematic influence on a paper's content, perceptions of conflict can erode trust in science as much as actual conflicts of interest. Ultimately, readers must be able to make their own judgments regarding whether an author's relationships and activities are pertinent to a paper's content. These judgments require transparent disclosures. An author's complete disclosure demonstrates a commitment to transparency and helps to maintain trust in the scientific process.

The corresponding author collects the conflict of interest disclosure from all authors. The corresponding author must include a summary statement in the manuscript in a separate section "Conflicts of Interest" placed just before the reference list. The statement should reflect all the collected potential conflict of interest disclosures in the form.

Journal Name:	Genes
Manuscript ID (if known):	genes-2607752
Manuscript Title:	Molecular Characterization of Complete Genome Sequence of a Recombinant Avian Coronavirus Identified in a Backyard Chicken from Tanzania
Authors:	Henry Muriuki Kariithi, Jeremy D. Volkening, Gaspar H. Chiwanga, Iryna V. Goraichuk, Peter L. M. Msoffe, David L. Suarez
Corresponding Author(s):	Henry Muriuki Kariithi, David L. Suarez

Financial interests

These may include but are not limited to membership, employment, consultancies, stocks/shares ownership; honoraria; grants or other funding; paid expert testimony and patent-licensing arrangements. Some examples are as follows:

- An author has received or expects to receive research grants from funding agencies (please provide details regarding the research funder and the grant number)
- An author has received support from commercial sources of funding by companies that sell drugs, medical devices, or provide medical services
- An author has received honoraria for speaking at symposia
- An author holds a position on advisory boards
- An author is an inventor of any planned, pending, or awarded patent on this work (please provide detailed information such as regarding the patent applicant, name of inventors, application number, status of application, specific aspects of manuscripts covered in the patent application)
- An author is an employee of an organization or company

Non-financial interests

These may include but are not limited to personal relationships or competing interests directly or indirectly tied to this research, or professional interests or personal beliefs that may influence your research.

The authors whose names are listed immediately below certify that they have NO conflicts of interest to declare:

Henry Muriuki Kariithi
Jeremy D. Volkening
Gaspar H. Chiwanga
Iryna V. Goraichuk
Peter L. M. Msoffe
David L. Suarez

The authors whose names are listed immediately below report the following conflicts of interest related to the work under consideration:

Not Applicable.

This statement is signed by all the authors to indicate agreement that the above information is true and correct (*a photocopy of this form may be used if there are more than 10 authors*):

Author's name (typed)	Author's signature	Date
Henry Muriuki Kariithi	Dr. Henry M. Kariithi Digitally signed by Dr. Henry M. Kariithi Date: 2023.08.31 01:00:40 +03'00'	August 31, 2023
Jeremy D. Volkening		
Gaspar H. Chiwanga	[signature]	08/31/2023
Iryna V. Goraichuk		
Peter L. M. Msoffe		
David L. Suarez		
